# Training and education in digital psychiatry: A perspective from Asia‐Pacific region

**DOI:** 10.1111/appy.12501

**Published:** 2021-12-07

**Authors:** Laura Orsolini, Chonnakarn Jatchavala, Isa Multazam Noor, Ramdas Ransing, Yuto Satake, Sheikh Shoib, Bigya Shah, Irfan Ullah, Umberto Volpe

**Affiliations:** ^1^ Unit of Clinical Psychiatry, Department of Clinical Neurosciences/DIMSC, School of Medicine Polytechnic University of Marche Ancona Italy; ^2^ Department of Psychiatry Prince of Songkla University Songkhla Thailand; ^3^ Department of Psychiatry YARSI University Jakarta Indonesia; ^4^ Department of Psychiatry BKL Walawalkar Rural Medical College Ratnagiri Maharashtra India; ^5^ Department of Psychiatry, Course of Integrated Medicine Osaka University Graduate School of Medicine Osaka Japan; ^6^ Department of Psychiatry Jawahar Lal Nehru Memorial Hospital (JLNMH) Srinagar Jammu and Kashmir India; ^7^ Department of Psychiatry Patan Academy of Health Sciences, School of Medicine Lalitpur Nepal; ^8^ Kabir Medical College Gandhara University Peshawar Pakistan

**Keywords:** Asia‐Pacific region, digital psychiatry, e‐mental health, medical education, psychiatry training, telemedicine, telepsychiatry

## Abstract

**Background:**

Digital mental health interventions and digital psychiatry have been rapidly implemented over the past decade, particularly with the intent to offer a cost‐effective solution in those circumstances in which the current mental health services and infrastructure are not able to properly accommodate the patients' needs. However, mental health workforce is often poorly theoretical/practical trained in digital psychiatry and in delivering remote consultations safely and effectively, not being common to own curricula‐specific training requirements in digital psychiatry and skills.

**Methods:**

A web‐based international cross‐sectional survey was carried out by a working group constituted by one or two national representative(s) of each WHO South‐East Asia and Western Pacific Regions (APAC), with the aim to evaluate the level of training, knowledge, experience, and perception regarding the topic of digital psychiatry in a sample constituted by medical students, psychiatry trainees, and early career psychiatrists from APAC.

**Results:**

An overall lack of theoretical and/or practical training on new digital tools and digital health interventions in psychiatry was observed. The level of training influences knowledge background, which, in turns, influences young professionals' perceptions and opinions regarding digital psychiatry and interventions in mental health.

**Conclusion:**

Implementing psychiatry training programs may significantly improve the level of knowledge and use of digital tools in mental healthcare. Moreover, mental health services and infrastructures should be properly adapted to the digital era, considering the overall weak and heterogeneous technical support and equipment, issues of internet connectivity, and other administrative‐related challenges observed in APAC.

## 1 INTRODUCTION

Digital health technologies typically refer to any form of remote/online platform or mobile device that can be used or designed to deliver a health intervention, including smartphone‐based apps, text messaging, telemedicine, telepsychiatry, wearable devices such as smart watches and online platforms and programs (Lipschitz et al., 2019; Wasil et al., 2020). Digital mental health interventions and digital psychiatry have been rapidly implemented over the past decade, particularly with the intent to offer a potential solution in those problematic circumstances and logistic issues for which the current mental health service infrastructure is not able to adequately accommodate to the needs of most patients (Husain et al., 2021; Lipschitz et al., 2019; Torous et al., 2019, 2020). In fact, digital psychiatry can be ideally widely disseminated with virtually no marginal cost, not requiring patient's transportation, and incentive patient autonomy, accessible in every moment when patient most needs support and with an efficacy comparable to traditional in‐person interventions, despite different existing country‐specific rules and laws in the field of digital psychiatry (Apaydin et al., 2018; Ng, 2020; Wells et al., 2018; Wright et al., 2019).

The need for implementing mental health services with digital psychiatry interventions mainly reside in the existing mental health care gap between the burden of mental disorders and the lack of appropriate resources and services necessary to treat them, particularly in the low‐income and middle‐income countries (LMICs) in which with up to 90% of people with mental disorders do not adequately receive treatment and follow‐up (Carter et al., 2021; Merchant et al., 2020; Pathare et al., 2018). The Asia‐Pacific (APAC) region comprises the part of the world in or near the Western Pacific Ocean, including east Asia (i.e., China, Hong Kong, Japan, Macau, Mongolia, North Korea, South Korea, Taiwan), south Asia (i.e., India, Bangladesh, Bhutan, Maldives, Nepal, Pakistan, Sri Lanka, Afghanistan, and British Indian Ocean Territory), southeast Asia (i.e., Brunei, Cambodia, Christmas Island, Cocos Islands, Indonesia, Laos, Malaysia, Myanmar, Philippines, Singapore, Thailand, Timor‐Leste, Vietnam), Australasia (i.e., Australia and New Zealand), and Oceania (i.e., Melanesia, Micronesia and Polynesia regions), including many LMICs. The World Health Organization (WHO) divides the APAC in two WHO regions, that is, South‐East Asia (i.e., Bangladesh, Bhutan, Democratic People's Republic of Korea, India, Indonesia, Maldives, Myanmar, Nepal, Sri Lanka, Thailand, and Timor‐Leste) and Western Pacific (i.e., Australia, Brunei Darussalam, Cambodia, China, Cook Islands, Fiji, Japan, Kiribati, Laos People's Democratic Republic, Malaysia, Marshall Islands, Micronesia, Mongolia, Nauru, New Zealand, Niue, Palau, Papua New Guinea, Philippines, Republic of Korea, Samoa Singapore, Solomon Islands, Tonga, Tuvalu, Vanuatu, and Vietnam). However, the APAC region is extremely vast and heterogeneous regarding the mental health system, cultural and ethnic background, geographic, and world bank income (WBI) features, and often the lack of awareness and adequate mental health service infrastructures addressed to those individuals with mental disorders may constitute a barrier to the access, care, treatment, and follow‐up (Knaak et al., 2017).

The digitalization of mental health care may offer direct support to individuals affected with mental disorders, by improving the quality of services provided and making evidence‐based interventions more widely available (Bhugra et al., 2017). In fact, the supply of mental health care may be hampered by the limited numbers of mental health professionals and inequitable distribution of providers in some countries belonging to the APAC region. For example, in India, there is approximately one trained psychiatrist for every 250 000 people and the total mental health workforce available is less than one provider per 100 000 people and many of them are mainly resident in major cities (Lahariya, 2018). In the Indian Kashmir region, there are around 41 psychiatrists and 12 clinical psychologists of a Kashmiri population of approximately 12.5 million (Shoib & Yasir Arafat, 2020a). The situation in other APAC countries may largely vary, despite the lack of specialty physicians in each remote area appearing to be a condition frequently present. Therefore, implementing and facilitating the digitalization of mental health services may represent an opportunity to enable individuals to access adequate mental healthcare in communities where mental health services may not be otherwise available and connecting patients with remote services delivered by non‐specialized providers (Carter et al., 2021; Pathare et al., 2018).

However, most mental health workforce does not own an appropriate theoretical either practical training in digital psychiatry and in delivering remote consultations safely and effectively, particularly among those coming from LMICs mainly due to technological and economical barriers encountered in applying digital psychiatry. Most countries do not have curricula‐specific training requirements, either at core or higher specialty level, for psychiatry trainees to demonstrate competence in digital skills that may be considered essential to good clinical practice, including abilities and competencies needed to provide and deliver mental health intervention by using digital tools (Bhugra et al., 2017; Dave et al., 2020).

The present study aimed to evaluate the level of knowledge, training, and experiences on digital psychiatry and related disciplines (e.g., e‐health, e‐mental health, telemedicine, telepsychiatry) in a cohort of medical students, psychiatry trainees, and early career psychiatrists (ECPs) coming from WHO APAC, in order to evaluate which needs and implementing strategies should be addressed in APAC countries to increase access to digital mental health interventions and care.

## 2 METHODOLOGY

### 2.1 Study design and sample recruitment strategy

A web‐based international cross‐sectional survey was carried out by using Google Form® in the timeframe from May 22, 2021 to July 23, 2021 by a working group constituted by one or two national representative(s) of each WHO South‐East Asia and Western Pacific Regions, with the aim to evaluate the level of training, knowledge, experience, and perception regarding the topic of digital psychiatry in a sample constituted by medical students, psychiatry trainees, and ECPs from APAC. National representatives were recruited through a link to a Google Form™ specifically designed to collect preliminary data (i.e., contact details including email address and affiliation, country/WHO region of residency and work, employment status, the average number of completed surveys able to be collected in a period of around 3–4 weeks) as well as the interest and availability in actively participating in the data collection. The link was disseminated within the WhatsApp® group of the ECPs of the World Psychiatric Association (WPA) in the timeframe from April 9, 2021 to May 20, 2021. The countries not included in the survey were those in which it was not possible to identify a national coordinator who would take over the responsibility of the study (e.g., Bhutan, Maldives, Myanmar, Timor‐Leste, Brunei, Darussalam, Cambodia, China, Cook Islands, Fiji, Kiribati, Lao People's Democratic Republic, Marshall Islands, Micronesia, Mongolia, Nauru, Niue, Palau, Papua New Guinea, Solomon Islands, Tonga, Tuvalu, Vanuatu, and Vietnam) or those countries in which the national coordinator (even though initially selected and invited to join the project, e.g., Bangladesh, Sri Lanka, Philippines, Malaysia, Republic of Korea, Singapore, Australia, and New Zealand) was unable to collect questionnaires for each of the above mentioned three categories from their own country. All respondents who met the following inclusion criteria have been included in the analysis of our study: (a) subjects belonging to the above‐mentioned categories (e.g., medical students, psychiatry trainees or ECP); (b) subjects belonging to one of the above‐mentioned WHO regions (i.e., South‐East Asia or Western Pacific Regions); (c) all subjects who agreed to participate to the study; (d) all subjects who authorized the treatment of sensible and personal data for research purpose. While all subjects who disagree to participate in the study or who did not fill out all sections of the survey have been removed by the dataset.

### 2.2 The structure of the survey

An ad hoc questionnaire constituted by four sections, was self‐administered anonymously to all subjects recruited in the present study, after asking to give informed consent as legally and ethically required. The first section included a set of sociodemographic data structured in 12 questions (of which seven multiple answer questions and five open‐ended questions). The second section was constituted by 17 questions (10 multiple answer, 4 open‐ended, and 3 questions with an increasing five‐item Likert scale), evaluating the level of training and education (if any) received by respondents regarding the following topics: e‐health, telemedicine, e‐mental health, telepsychiatry, digital psychiatry, and digital health interventions (DHIs). The third section was referred to the general level of knowledge on telemedicine, telepsychiatry, and digital psychiatry and is constituted of 18 items in which each question gave zero point (if wrongly answered), 1 point (when correctly answered) and each value from zero to one in those cases in which more than one answer is possible for that question. The third section also included one question (C8) which is a multiple answer question. The sum of each item except C8 was built to create a continuous variable named knowledge score (*K*) ranging from 0 to 18. The fourth section was made of 33 questions, of which 29 questions with a dichotomous answer and 4 questions with an increased five‐item Likert scale, to investigate the participants' opinions, experiences, and perceptions regarding the telemedicine, telepsychiatry, and digital psychiatry.

### 2.3 Data collection

National representatives facilitated the delivery of the English version of the survey across all APAC countries, through an online data collecting system. No translation in other languages was deemed necessary, as participants were deemed by their national representatives to have sufficient command of English to reliably answer the questions. Among South‐East Asia and Western Pacific Regions invited to take part in the survey, the following countries actually participated to our survey: India, Indonesia, Japan, Nepal, Pakistan, and Thailand.

### 2.4 Statistical analysis

A preliminary descriptive analysis was carried out by using the *Software Statistical Package for Social Sciences* (SPSS) for *MacOS* (version 26.0, IBM Corp., Armonk, NY). All categorical variables were summarized as frequencies (*n*) and percentages (%), while all continuous variables have been summarized as means (*m*) and standard deviations (*SD*) or median (*M*) and 95% confidence interval (CI), where appropriate. Pearson' *χ* ^2^ test was used to compare sociodemographic features and categorical variables, such as the level of training in digital psychiatry. The normality of the *K* score was confirmed by using the Kolmogorov–Smirnov and Shapiro–Wilk normality tests. Independent student's *T*‐test and two‐tailed Mann–Whitney test, were performed, when appropriate, to compare *K* scores according to the following dichotomous variables: gender, WHO Region (residency), WHO Region (birthplace), opportunity to have received a practical/theoretical training and to have applied it in the clinical practice (B22 item), modules/topics of digital psychiatry taught within the Faculty of Medicine and Surgery (from B26 to B31 items) or within the psychiatry training program (from B32 to B37 items) and all dichotomic items of section regarding participants' attitudes and beliefs regarding digital psychiatry‐related contents (from D1 to D22 and from D28 to D34). The analysis of variance (ANOVA) was used to perform all comparisons between the groups identified through *K* scores with respect to the main socio‐demographic characteristics (i.e., marital status, country residency, country of origin, ethnicity, WBI, current professional/academic role, country of Medicine Faculty, country of psychiatry training program, working country as psychiatrist). Moreover, ANOVA was performed to compare *K* scores and the level of participants' training and opinion regarding the efficacy of digital interventions versus the efficacy of face‐to‐face modality. The significance level was set a priori at *p* ≤ .05, two‐tailed.

## 3 RESULTS

The survey was filled out by 221 respondents, 5 of which were excluded in the analysis due to their subsequent refusal to participate in the study, and 24 as they did not belong to WHO South‐East Asia or Western Pacific Regions. The total number of questionnaires correctly filled during the collection process and finally included into the downstream analysis was of 192.

### 3.1 Sample characteristics

The final sample included 192 participants, of which 69 females (35.9%) and 123 males (64.1%). The average age of respondents is 27.5 (±*SD* = 5.4) years, with an average number of years practicing psychiatry of 2.1 (±*SD* = 2.4, CI% 1.8–2.5). Most respondents declared to be single (never married; *N* = 130, 67.7%) and Asian as ethnicity (*N* = 172, 89.6%). The sample was constituted mainly by respondents who declared to be resident in WHO South‐East Asia Region (*N* = 184, 95.8%), being India (*N* = 60, 31.3%), Nepal (*N* = 59, 30.7%), and Thailand (*N* = 38, 19.8%) the most represented countries of residency, even though all respondents declared they were born in one of the countries belonging to the WHO South‐East Asia Region (*N* = 192; 100%). Most respondents declared to live in a large city/town (over 500 000 population) or in a medium city/town (10 000–500 000 population), being reported in 48.4% (*N* = 93) and in 30.7% (*N* = 59) of the sample, respectively. Participants mainly declared to own from a lower middle (*N* = 92, 47.9%) to an upper‐middle (*N* = 61, 31.8%) annual WBI, even though most participants coming from countries classified as LMICs (*N* = 179; 93.2%). The sample is mainly represented by medical students (*N* = 104, 54.2%), even though there is a percentage of 21.4% of ECPs, 19.3% of psychiatry trainees and 5.2% of medical doctors (M.D.) waiting for starting psychiatry training program. Mainly respondents declared to have studied medicine in India (*N* = 55, 28.6%), Nepal (*N* = 52, 27.1%), and Thailand (*N* = 40, 20.8%), followed by Pakistan (*N* = 20, 10.4%) and Japan (*N* = 13, 6.8%). Similarly, most respondents declared to attend or have attended their psychiatry training program in India (*N* = 59, 30.7%) and Nepal (*N* = 48, 25.0%), followed by Thailand (*N* = 38, 19.8%), Pakistan (*N* = 17, 8.9%), and Japan (*N* = 13, 6.8%; Table 1).

**TABLE 1:** Sociodemographic characteristics of the sample

	*N* (%)	
Gender	Male	123 (64.1%)
	Female	69 (35.9%)
Marital status	Single (never married)	130 (67.7%)
	Married or co‐living partner	38 (19.8%)
	In a stable affective relationship	24 (12.5%)
Country of residency	Indonesia	5 (2.6%)
	Japan	13 (6.8%)
	Pakistan	17 (8.9%)
	Thailand	38 (19.8%)
	Nepal	59 (30.7%)
	India	60 (31.3%)
Living city	Village/rural	17 (8.9%)
	Small city/town (10 000–100 000 population)	23 (12%)
	Medium city/town (100 000–500 000 population)	59 (30.7%)
	Large city/town (over 500 000 population)	93 (48.4%)
Born country	South Korea	1 (0.5%)
	Indonesia	6 (3.1%)
	Japan	12 (6.3%)
	Pakistan	17 (8.9%)
	Thailand	38 (19.8%)
	Nepal	57 (29.7%)
	India	61 (31.8%)
Ethnicity	Caucasian	5 (2.6%)
	Asian	172 (89.6%)
	Mixed	15 (7.8%)
World Bank Income	Low	30 (15.6%)
	Lower‐middle	92 (47.9%)
	Upper‐middle	61 (31.8%)
	High	9 (4.7%)
Current academic role	Medical students	104 (54.2%)
	Medical doctors waiting for starting psychiatry training program	10 (5.2%)
	Psychiatry trainees	37 (19.3%)
	Early career psychiatrists	41 (21.4%)
Country of medical college	China	2 (1%)
	Bangladesh	2 (1%)
	Australia	2 (1%)
	Indonesia	6 (3.1%)
	Japan	13 (6.8%)
	Pakistan	20 (10.4%)
	Thailand	40 (20.8%)
	Nepal	52 (27.1%)
	India	55 (28.6%)
Country of psychiatry residency	USA	1 (0.5%)
	Australia	2 (1%)
	Indonesia	6 (3.1%)
	Japan	13 (6.8%)
	Thailand	38 (19.8%)
	Nepal	48 (25%)
	India	59 (30.7%)

### 3.2 Level of training and education

Most respondents reported that they did not receive a dedicated teaching course within their Faculty of Medicine and Surgery on e‐health (*N* = 128, 66.7%), telemedicine (*N* = 110, 57.3%), e‐mental health (*N* = 124, 64.6%), telepsychiatry (*N* = 111, 57.8%), digital psychiatry (*N* = 138, 71.9%), DHIs (*N* = 143, 74.5%). Almost half of respondents reported that telepsychiatry has never been taught within their psychiatry course at the Faculty of Medicine and Surgery (*N* = 95, 49.5%), while 26% of the sample (*N* = 50) declared that it was taught very little (less than 20% of total psychiatry teaching). Only 6.8% of the sample (*N* = 13 respondents) declared to have received enough teaching in telepsychiatry (61%–80% of total teaching) within their psychiatry course during the Faculty of Medicine and Surgery. Moreover, more than half of respondents overly reported that they did not receive a dedicated teaching course within their psychiatry training program on e‐health (*N* = 129, 67.2%), telemedicine (*N* = 114, 59.4%), e‐mental health (*N* = 124, 64.6%), telepsychiatry (*N* = 99, 51.6%), digital psychiatry (*N* = 126, 65.6%), DHIs (*N* = 137, 71.4%). Furthermore, even when it was asked to participants which is the level of theoretical training in telepsychiatry they received within their psychiatry training program, almost half of the sample reported that they did not receive it (*N* = 89, 46.4%). According to the country‐level responses, the lack of theoretical training in telepsychiatry was reported in 92.3% of Japanese respondents, 58.8% of Pakistani respondents, 50% of Thailand, and 45.8% of Nepal respondents. While 31.8% of the total sample declared to have received it very little (less than 20% of total psychiatry teaching), being represented by 60% of Indonesian respondents and 39% of Nepal respondents. Overall, a significant higher percentage of respondents who reside in Nepal declared a lack or poor theoretical training in digital psychiatry, compared to other countries [*χ* ^2^(20) = 33.682, *p* = .028]. Only 6.3% of the sample (*N* = 12) declared they were offered theoretical training in telepsychiatry during their psychiatry training course. Similarly, respondents declared to have never received a practical training in the field of telepsychiatry within their psychiatry training program is represented by 50.5% of the total sample, whereas 27.1% of the total sample reported a poor practical training in telepsychiatry (i.e., less than 20% of total psychiatry training program). Despite this condition, most respondents supported the need to implement digital psychiatry and related topics within a course/teaching module within the Faculty of Medicine and Surgery, being reported as rather important in 35.9% (*N* = 69) and very important in 45.8% (*N* = 88) of respondents. Slightly higher percentages were reported among respondents when they were asked their opinion regarding implementing telepsychiatry within the psychiatry training program (respectively, with 37% of respondents who replied that it would be rather important and 47.9% who answered that it would be very important). Similarly, it was declared by respondents for other digital related topics, such as digital health interventions (78.6% and 80.2%), digital psychiatry (79.7% and 82.8%), e‐health (80.8% and 81.7%), e‐mental health (82.9% and 83.4%), telemedicine (82.3% and 79.7%), when it was asked, respectively, their opinions regarding the need to implement them within the Faculty of Medicine and within their psychiatry training program. Respondents who declared to have acquired a theoretical and/or a practical training in some digital related disciplines (e.g., e‐health, e‐mental health, telemedicine, telepsychiatry, digital psychiatry, DHIs) within the medical school and/or psychiatry training program, declared that they had the opportunity to use and apply this acquired knowledge in their clinical practice is represented by 64.1% of cases. Among these respondents, 29.3% (*N* = 36) reported a moderate use (average one to two times monthly), 22.0% (*N* = 27) a frequent use (one to two times a week), and an occasional use (less than one time monthly) in 19.5% (*N* = 24). While among these respondents (*N* = 123), it was mainly reported an occasional use (less than one time monthly) in 24.4% (*N* = 30) or a rare use (less than one to two times annually) in 22.8% (*N* = 28) of them, when it was asked if their usage was provided before the COVID‐19 pandemic. At this regard, 35.8% of this subsample (*N* = 123) declared that the current COVID‐19 pandemic moderately intensified (i.e., by determining an increase from 21% to 50% compared to the previous clinical practice) the use of digital psychiatry interventions (e.g., e‐health, e‐mental health, telemedicine, telepsychiatry, digital psychiatry, DHIs) in their clinical practice.

### 3.3 Level of knowledge

**FIGURE 1:**
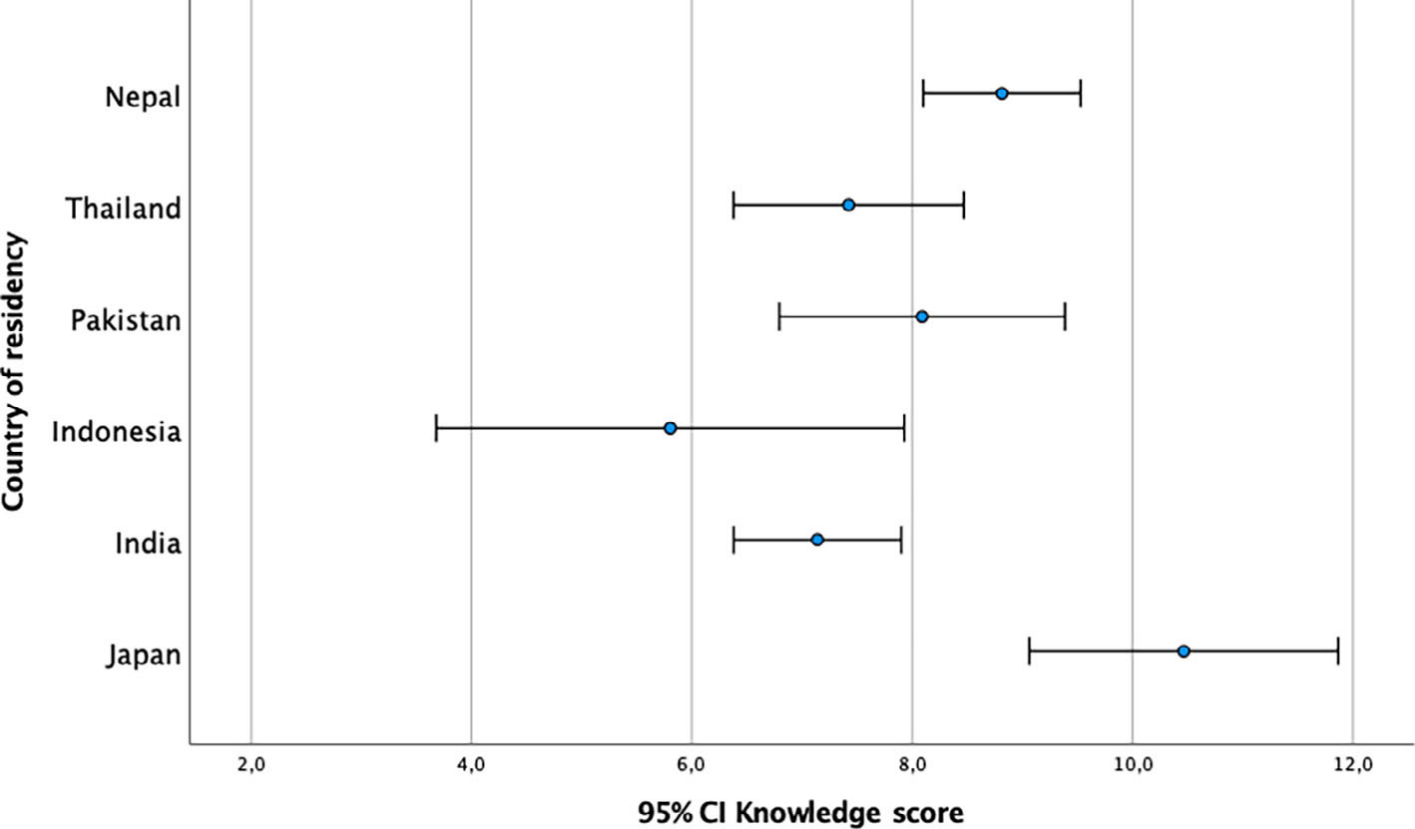
Knowledge scores across countries of residency

**FIGURE 2:**
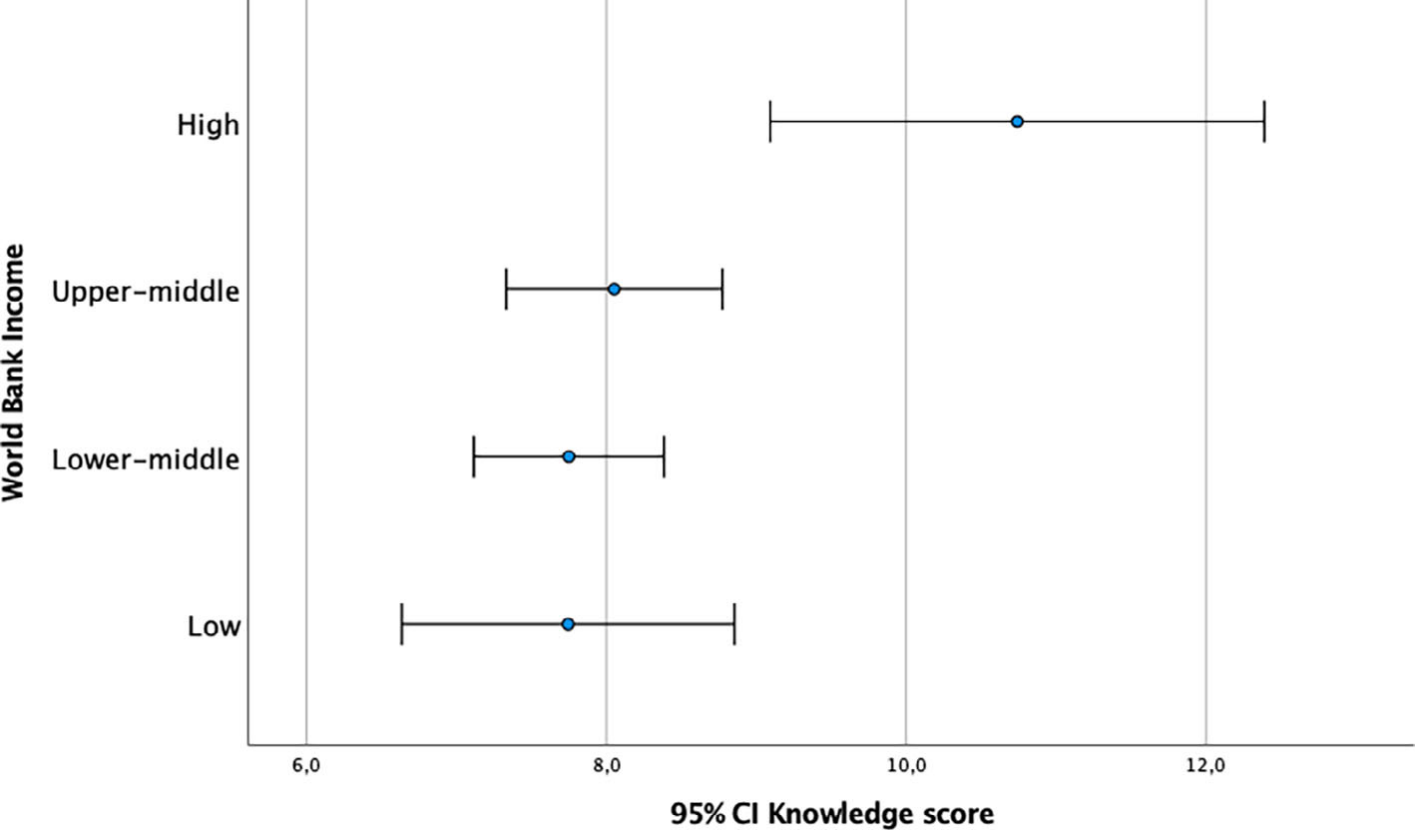
Knowledge scores across countries, according to the World Bank Income

**FIGURE 3:**
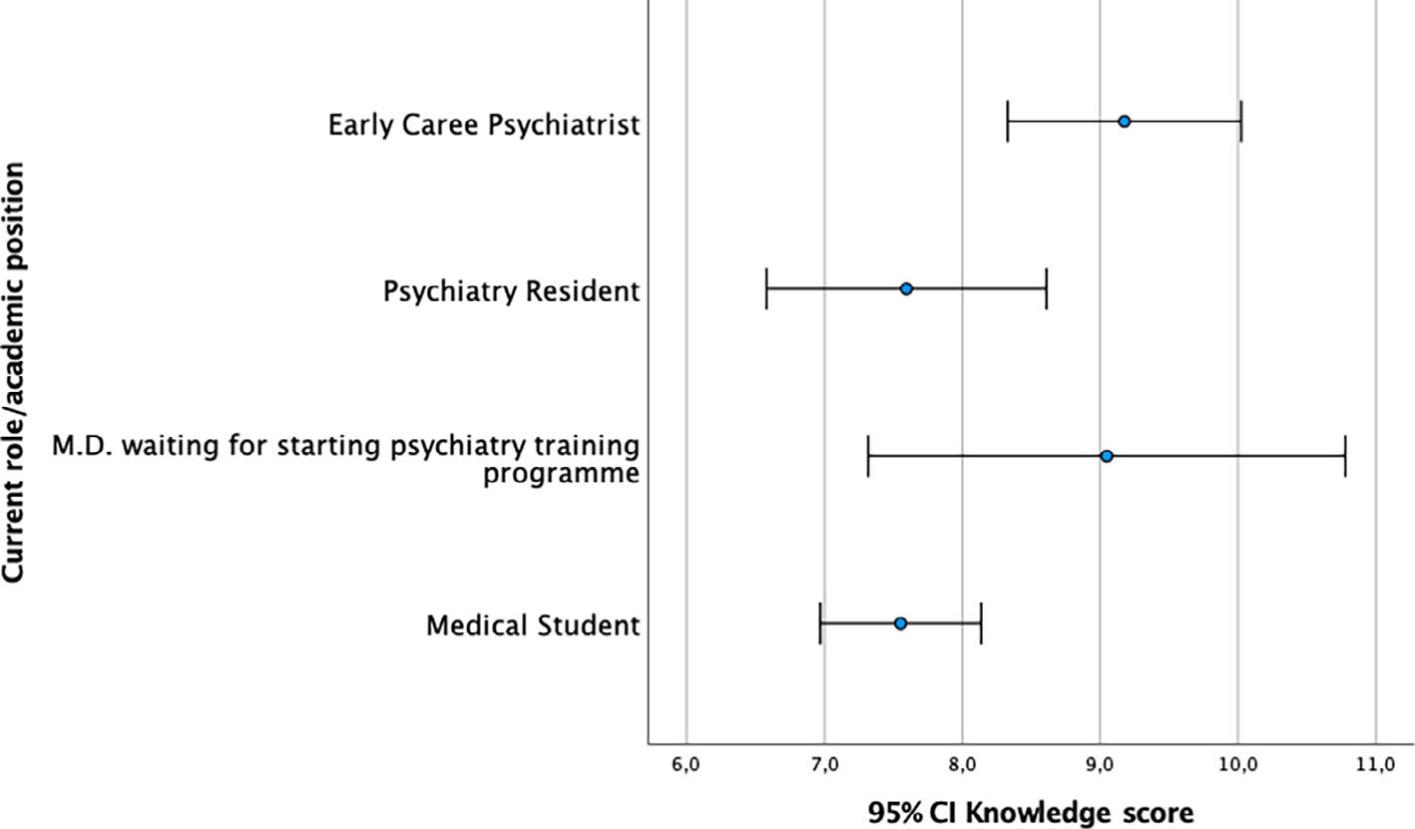
Knowledge scores according to academic role/position

The average mean *K* score was 7.9 (±*SD* = 2.9), with statistically significant higher scores among participants resident in WHO western pacific region compared to WHO south‐east Asia region (*t*(190) = −2.088, *p* = .038). Participants resident in Japan reported significantly higher *K* scores compared to India (*p* = .002), Indonesia (*p* = .025), and Thailand (*p* = .013). Participants resident in Nepal reported significant higher *K* scores compared to India (*p* = .019; Figure 1). Participants who declared a high WBI reported significantly higher *K* scores compared to lower middle (*p* = .021) and low income (*p* = .039; Figure 2). ECPs reported significant higher *K* scores compared to medical students (*p* = .015; Figure 3). Participants who attended the Faculty of Medicine in Japan reported significantly higher *K* scores, compared to those who studied in India (*p* = .006), Indonesia (*p* = .009), and Thailand (*p* = .030). Participants who studied in Nepal reported significant higher *K* scores compared to those studied in India (*p* = .038). Participants who studied their psychiatric training program in Japan also reported higher *K* scores compared to other countries (*p* = .004). Participants who currently work in Japan reported significant higher *K* scores compared to other countries (*p* < .001). Those respondents who declared that digital psychiatry and related digital tools may ensure the equity of access to mental health care [*t*(190) = 2.512, *p* = .013] and those who declared that digital psychiatry may ensure greater availability of qualified mental health care in remote areas [*t*(190) = −3.290, *p* = .001] reported significantly higher *K* scores. Participants who reported that digital psychiatry may improve mental health care in disadvantages contexts (e.g., prison, emergency situation, etc.) and quality of mental health care by ensuring continuity of care, reported significantly higher *K* scores [*t*(190) = −2.486, *p* = .014; and, *t*(190) = −2.857, *p* = .005, respectively]. Similarly, those participants who declared that digital psychiatry is able to improve the quality of mental health care for patients with chronic diseases [*t*(190) = −3.045, *p* = .003], to reduce the mobility for patients resident in remote areas [*t*(190) = −2.450, *p* = .015] and patients' waiting time [*t*(190) = −2.717, *p* = .007] reported significant higher *K* scores. Moreover, those participants who declared that digital psychiatry is able to facilitate the building of a valid therapeutic alliance [*t*(190) = −2.156, *p* = .032] and ensure the continuity of mental health care during the current COVID‐19 pandemic [*t*(190) = −3.994, *p* < .001] reported significant higher *K* scores. Only 46.4% (*N* = 89) of the sample was able to correctly provide a definition of e‐health, being the most educated countries Japan (53.8% of Japanese respondents), Pakistan (64.7% of Pakistani respondents), and Nepal (62.7% of Nepali respondents) [*χ* ^2^(5) = 23.331, *p* < .001] and those who attended their psychiatry training program in Japan and in Nepal [*χ* ^2^(7) = 27.858, *p* < .001].

### 3.4 Level of experience and attitudes towards digital psychiatry

Around 81.3% of participants (*N* = 156) believe that digital psychiatry might ensure equity of access to mental health care while 87.5% (*N* = 168) a greater availability of qualified mental health care professionals in remote areas, without any significant differences across countries. Moreover, 83.3% (*N* = 160) of the sample declared that digital psychiatry may improve mental healthcare in disadvantaged contexts or circumstances which imply additional travel costs or during emergency management (e.g., in prison), without any significant differences across countries. Most participants (84.4%, *N* = 162) declared that digital psychiatry is able to provide drug therapy support by improving treatment adherence and patients' compliance, without any significant differences across countries. Overall, 82.3% (*N* = 158) of participants believe that digital tools own the potential to guarantee a more rationalization of social‐health‐financial processes with a possible cost saving, including the reduction of hospitalization (85.4%; *N* = 164), travel (87%; *N* = 167), and patients' waiting list (83.3%; *N* = 160), and relative costs (81.3%; *N* = 156), without any significant country‐based differences. Overall, most participants (77.1%; *N* = 148) believe that digital psychiatry interventions are able to provide interventions comparable to those traditional in‐person ones in terms of efficacy, effectiveness, and utility, without any significant differences across countries. Regarding privacy and safety concerns, most respondents (73.4%; *N* = 141) agreed that digital psychiatry is able to ensure adequate safety, privacy, and data protection. In addition, most sample (84.4%; *N* = 162) declared that digital psychiatry interventions may allow clinicians to build a valid therapeutic alliance despite the digital modality and ensure continuity of care in times of emergencies, such as the current COVID‐19 pandemic (87%; *N* = 167), particularly for those vulnerable people (85.9%; *N* = 165) including elderly (76%; *N* = 146). Around one out of three participants (68.2% of the sample; *N* = 131) believe that digital psychiatry may replace face‐to‐face interventions. Regarding technical and clinical issues, participants trained in Japan, India, Pakistan, Thailand, and Nepal significantly declared the need to preliminarily acquire an accurate and balanced assessment between risks versus benefits/contraindications of digital psychiatry interventions in specific categories of patients, before offering the service [*χ* ^2^(5) = 14.314, *p* = .014]. Most participants believe that digital psychiatry may be indicated mainly for follow‐up visits of already known and pharmacologically stable patients (86.5%; *N* = 166) and it is effective as face‐to‐face interventions (65.9%; *N* = 126), without any country differences. Participants trained in Nepal, Pakistan, Indonesia, and Japan significantly declared that doctors should acquire a preliminary theoretical and practical training to be considered capable in providing therapeutic interventions through technological devices [*χ* ^2^(5) = 14.733, *p* < .012].

## 4 DISCUSSION

To the best of our knowledge, this is the first study collecting data on training, education, and knowledge on digital psychiatry in a cohort of young mental health professionals and medical students from APAC countries. Despite our findings having been collected by both WHO South‐East Asia and Western‐Pacific regions, only Japan is representative of the WHO Western‐Pacific region. Countries coming from WHO South‐East Asia region include India, Nepal, Indonesia, Thailand, and Pakistan. Overall, our findings indicate a poor or a lack of an adequate (both in terms of contents and dedicated teaching time) theoretical and practical training in digital psychiatry and related topics disciplines (e.g., e‐health, e‐mental health, telemedicine, telepsychiatry) across different countries here included, which is in line with previous studies (Casà et al., 2021; Cory & Stevens, 2020; Feroz et al., 2021; Jameson et al., 2011; Pinto da Costa et al., 2019; Pote et al., 2021; Tudor Car et al., 2021). However, despite the initial intent was recruiting both LMICs and upper‐middle‐income countries to compare findings and estimate whether WBI may influence the level of training, experience, and knowledge on digital psychiatry, our sample is mainly constituted by six countries, of which only Japan is included in upper‐middle‐income countries, according to the gross national income per capita as published by the World Bank (World Bank Data, 2021). However, participants from Japan are an extreme limited number which does not allow to do a comparison analysis. However, one could argue that our sample is extremely represented by LMICs which display interesting findings and country‐based variability, despite the same average level of WBI. The 2019 Global Digital Health Index reported that most LMICs need digital health training to meet current demand in mental health care and treatment, by underlining as the current digital health maturity phase (scored from 1 to 5, from the least mature to the most mature digitalization process) for Pakistan is equal to 2 and for Indonesia is equal to 3, whereas only a few LMICs, including Thailand showed promising results in creating specialized digital health workers, being scored as at fourth stage of digital maturation (Global Digital Index, 2019; Kapoor et al., 2020). The poor training appears to reflect a general poor knowledge of participants in this field of psychiatry which, in turns, may determine a poor attitude and false beliefs regarding digital tools and interventions in terms of their evidence‐based efficacy and effectiveness as well as their advantages, target population, and target mental health disorders. In fact, as already demonstrated by previous published studies, clinicians' knowledge may greatly influence their attitudes to offer and recommend digital psychiatry interventions and, in turns, indirectly influence patients' perceptions and choice of digitally driven mental health care (Gibson et al., 2011; Jameson et al., 2011; Whitten & Mackert, 2005).

Despite the limited training opportunities received, most participants declared the great need to implement digital psychiatry and related disciplines (e.g., e‐health, e‐mental health, telemedicine, telepsychiatry) since medical school and, subsequently, in dedicated modules/courses within psychiatry training program. In particular, the topic mainly suggested by participants to be included as an essential tool is telepsychiatry. Obviously, this increasing perceived need by clinicians may be also determined by the current COVID‐19 pandemic and necessary restrictive measures and adaptations needed in the mental health services and infrastructures which indeed forced clinicians to reduce or discontinue in‐person consultations (Unützer et al., 2020), access to mental health care and services by patients, despite an increasing demand and request for de novo psychiatric onset due to the COVID‐19‐related situation (Chen et al., 2020; D'Agostino et al., 2020; Fagiolini et al., 2020; Fiorillo et al., 2020; Giallonardo et al., 2020; Gorwood & Fiorillo, 2021; Li et al., 2020; McIntyre & Lee, 2020; Rojnic Kuzman et al., 2021). In fact, accordingly, our findings reported a moderate increase in the frequency use of digital psychiatry interventions (e.g., e‐health, e‐mental health, telemedicine, telepsychiatry, digital psychiatry, DHIs) by participants who reported an elevation up to 21%–50% compared to their previous clinical practice, after the COVID‐19 outbreak (Stewart & Appelbaum, 2020). These findings are consistent with previous published studies carried out in India and Nepal (Li et al., 2021; Parikh et al., 2021; Rojnic Kuzman et al., 2021; Singh, 2021; Singh et al., 2021).

Furthermore, our sample showed an average *K* score very low which may be partially explained by the higher percentage of medical students recruited (54.2%), followed by ECPs (21.4%) and psychiatry trainees (19.3%). Therefore, we could argue that the lower *K* scores may reflect the fact that most participants have not already received their training in digital psychiatry and related disciplines, due to their medical student status and, hence, are not still able to correctly answer questions related to digital psychiatry and related disciplines. Moreover, this sample distribution may also explain the low percentage of subjects who declared to have not received a theoretical and/or practical teaching course in digital psychiatry and related disciplines (e.g., e‐health, e‐mental health, telemedicine, telepsychiatry) both within their medical school and psychiatry residency (Nagendrappa et al., 2021). However, in those participants who declared to have received some theoretical and/or practical training in digital psychiatry, even though at minimum extent, it was reported a higher possibility or likely a more positive attitude in applying it in their clinical practice. This evidence may also underline how extremely important is receiving, even though only at a basic level, digital skills and implementing digital competencies in the field of digital psychiatry as this may reflect a more positive attitude towards a digitally based clinical practice in mental health. In fact, in our sample, those who were trained in delivering digital interventions declared to be more ready and prone to apply them in their clinical practice.

Moreover, the levels of knowledge are not only influenced by the level of training and education in digital psychiatry but also by economic status, being higher among participants who own higher WBIs, probably due to the highest financial possibilities which may have individuals in buying digital tools and acquiring a better and more qualified education and training in the field of digital psychiatry. Moreover, despite the great differences in terms of sample size between participants belonging to the WHO South‐East Asia Region versus WHO Western Pacific Region, significant higher K scores were observed in Japanese participants compared to Indian, Indonesia, and Thailand participants. Moreover, within WHO South‐East Asia Regions, Nepal participants presented significantly higher K scores compared to Indians. This is partially being explained by geographically and economically significant differences in different Indian regions and, hence, in the implementation of digitalization of medicine and mental health services, particularly due to the fact that the Indian sample is mainly here represented by participants coming from rural‐remote regions and Kashmir region. For example, in some Indian regions, like Kashmir, the implementation of digital psychiatry in mental health service infrastructures may be challenging due to the second generation (2G) mobile network, frequent communication blackouts, poor digital literacy, and absence of a skilled, adequately educated and sufficient mental health workforce able to encounter the burden of Indian mental diseases (Shoib & Arafat, 2021). Therefore, in the Indian sample there is a great disparity in terms of Internet and digital tools access, as already reported in previous studies (Andersson et al., 2019; Firth et al., 2019; Ransing et al., 2021; Shoib & Yasir Arafat, 2020b). Moreover, most of the sample recruited by Nepal coming from Kathmandu city, which represents the capital of the country and one of the biggest and digitalized city of Nepal, being the city with more Internet access and connectivity as well as with more available digital psychiatry platforms (commercial; Singh et al., 2021). Moreover, most medical students coming from Nepal were recruited by the only medical college providing a telepsychiatry service for which medical students are trained and informed. Therefore, these findings may confirm the hypothesis that implementing training in digital psychiatry, including telepsychiatry, since medical school may significantly increase the level of knowledge and, hence, the general participants' opinions and beliefs regarding digital psychiatry and interventions in mental health. Moreover, studying medicine in Japan is associated with higher *K* scores, compared to India, Indonesia, and Thailand. Moreover, studying medicine in Nepal is associated with higher *K* scores compared to India. At this regard, the highest *K* scores of Japanese participants may also reflect the gap of countries' WBI, as Japanese participants may own better resources for digital psychiatry compared to other participants coming from LMICs. In addition, participants attending psychiatry resident programs and/or currently working in Japan show significantly higher *K* scores, compared to WHO South‐East‐Asia countries.

Furthermore, our findings indicate that the level of knowledge is also influenced by general participants' attitudes and beliefs towards digital psychiatry interventions in delivering mental health care and treatment, in terms of efficacy, effectiveness, safety, and privacy concerns as well as financial and administrative advantages. In fact, those participants who are less prone to have a good opinion regarding the potentialities and advantages of digital tools and more likely declare that digital interventions are less effective compared to traditional in‐person ones, showed lower levels of knowledge in the field of digital psychiatry. Despite these considerations, however, our findings indicate that most sample mainly believes that digital psychiatry may improve mental healthcare in disadvantages contexts or circumstances which imply additional travel costs or during emergency management (e.g., in prison), ensure equity of access to mental health care, a greater availability of qualified mental health care professional in remote areas and provide drug therapy support by improving treatment adherence and patients' compliance (Apaydin et al., 2018; Feijt et al., 2020; Gibson et al., 2011; Turvey et al., 2013; Wells et al., 2018; Wright et al., 2019). Moreover, regarding the COVID‐19 pandemic, most of the sample declared that digital psychiatry may potentially allow clinicians to ensure continuity of care in times of COVID‐19‐related emergencies, as already reported in other studies, particularly for more vulnerable and physically proven individuals, such as elderly (Chen et al., 2020; Feijt et al., 2020; Ghebreyesus, 2020; Hilty et al., 2013; Jameson et al., 2011; Wagnild et al., 2006). Furthermore, most of the sample declared advantages of digital psychiatry tools also in terms of financial and economic savings, by allowing a better rationalization of socio‐health‐financial processes, in terms of hospitalization rate reduction, travel cost reduction, and optimization of patient's waiting list. Therefore, digital psychiatry may potentially provide an advantage from an administrative and logistic perspective, especially to mental health infrastructures and national health organizations which are poorly organized or missing in the health workforce and specialty professionals. Moreover, in terms of efficacy and effectiveness, most participants believe that digital psychiatry interventions are comparable to those in‐person ones and may potentially replace them, particularly in times of emergencies, even though most participants suggested that digital psychiatry should be preferred mainly to those pharmacologically stable patients and those who need follow‐up visits. According to the respondents, digital psychiatry should not be recommended to those patients at their first consultation visit whereas it should be recommended a more traditional in‐person consultation, as already previously underlined by a study (Gibson et al., 2011). In addition, most participants of our study did not report consistent findings regarding concerns and issues on privacy, data protection, and safety of digital tools in mental health, having our sample mainly reported an overall good opinion of digital tools also in these privacy and safety‐related aspects, despite a previous study reported a negative perception by interviewed clinicians (Hale & Kvedar, 2014). However, regarding technical and clinical issues, participants mainly declared the need to preliminarily acquire an accurate and balanced assessment between risks versus benefits/contraindications of digital psychiatry interventions in specific categories of patients, before offering the service. This evidence is in line with the American Telemedicine Association (ATA) guidelines (Turvey et al., 2013) which recommended to preliminarily identifying a supporting caregiver who should be called and alerted by the clinician in case of emergency during a digital intervention and/or consultation. According to most of our sample, the identified caregiver should be warmly included in a structured and prevently agreed balanced plan for the management of a patient's crises during a digital consultation and/or intervention. Interestingly, despite not necessarily being a board‐certified training and certification regarding digital competencies in mental health, most participants believe that clinicians should firstly obtain a certified theoretical and practical training in digital psychiatry, before allowing them to deliver a digital intervention and consultation in mental health.

Despite this promising evidence, our study presents a set of limitations as listed below. First, the sample size not being equally distributed (in terms of sample size, sample features, percentage of participants for each category, etc.) according to included countries and not enlisting all countries belonging to the APAC Regions, may not be representative of the APAC situation. Moreover, WHO Western‐Pacific Region is only represented by Japan, which has a WBI averagely higher compared to other countries here included and has a different stage of digitalization of medicine disciplines. Secondly, the sample is mainly constituted by medical students who may not be completely aware about the situation in terms of training (if provided and to which extent) in the psychiatric residency training program. Thirdly, the cross‐sectional study design may allow us to have only the current situation of the sample, without considering the evolution over the time in terms of level of education and training (i.e., before vs. after COVID‐19 pandemic, over the progression towards psychiatry training program and after obtaining psychiatric specialization), the level of knowledge across the time (i.e., depending on the stage of academic position, the level of experience and clinical practice in the field of digital psychiatry, the stage of country digitalization in that specific area of health, etc.) and the general attitudes and beliefs (i.e., before and after a dedicated theoretical and/or practical training program in the field of digital psychiatry). Fourthly, the findings were presented may be potentially influenced by selection bias, particularly in those countries not enough English‐skilled, as the survey has been disseminated only in English. Fifthly, the cross‐sectional nature of study design does not allow comparing a group constituted by those who received a theoretical and/or practical training versus a group constituted by those who did not received it and how their level of knowledge and attitudes may change over the time before and after their clinical practice in digital health interventions.

Therefore, further research and more longitudinal and case–control study designs are needed to evaluate the effect of COVID‐19 pandemic on the level of education and training in digital psychiatry and related disciplines across APAC countries and how this may be influenced according to the different stages of academic progression and country digitalization. Moreover, further country‐specific national‐based studies should be carried out, and compare their findings to evaluate which is the best education and training strategy to ensure an adequate and homogeneous training in digital psychiatry and related disciplines across APAC countries, particularly those belonging to the LMIC. In fact, our findings demonstrated significant differences in those LMICs which implemented digitalization such as Nepal, in terms of education, training, and frontline experiences in applying digital psychiatry and related disciplines (e.g., e‐health, e‐mental health, telemedicine, telepsychiatry), by supporting the idea that incentivizing medical and psychiatry training programs in this field may potentially facilitate the digitalization process in LMICs.

## Data Availability

Data sharing is not applicable to this article as no new data were created or analyzed in this study.
